# Quality of Stroke Patient Information Applied in Randomized Controlled Trials—Literature Review

**DOI:** 10.3389/fneur.2020.526515

**Published:** 2020-12-07

**Authors:** Anna C. Alegiani, Anne C. Rahn, Anke Steckelberg, Götz Thomalla, Christoph Heesen, Sascha Köpke

**Affiliations:** ^1^Department of Neurology, University Medical Center Hamburg-Eppendorf, Hamburg, Germany; ^2^Department of Health Services Research, Carl von Ossietzky University Oldenburg, Oldenburg, Germany; ^3^Institute of Neuroimmunology and Multiple Sclerosis (INIMS), University Medical Center Hamburg-Eppendorf, Hamburg, Germany; ^4^Institute for Health and Nursing Science, Medical Faculty, Martin Luther University Halle-Wittenberg, Halle, Germany; ^5^Institute of Nursing Science, University of Cologne, Faculty of Medicine and University Hospital Cologne, Cologne, Germany

**Keywords:** stroke, prevention, knowledge, evidence based patient information, stroke patient information

## Abstract

**Background:** Strokes have a huge impact on patients' quality of life. Although there are potentially effective secondary preventions and treatment options for stroke patients, adherence is mostly low. Low disease and treatment-related knowledge and, consequently, a lack of informed decision-making in stroke patients may contribute to this problem. However, stroke patient information did not seem to have relevant effects on patients' knowledge in randomized controlled trials. *One* contributing factor may be the lack of thoroughly developed patient information materials.

**Methods:** We aimed to evaluate the quality of patient information materials for stroke patients by using randomized controlled trials, applying quality criteria for evidence-based patient information (EBPI). We conducted a literature search (MEDLINE, Embase, CINAHL, PsycINFO, and CENTRAL). To be included in the review, research had to be randomized controlled trials that provided stroke patient information, were published in English, and had knowledge assessed as the primary endpoint. Authors of primary studies were contacted and asked for information materials applied.

**Results:** We screened 15,507 hits and identified 30 eligible studies. Information materials were available for only eight studies. Analyses revealed that all available materials had important shortcomings concerning EBPI quality criteria [concerning, for example, structural information (e.g., reporting conflicts of interest), content information (e.g., reporting sources of information), or comprehensive descriptions of treatment effects and side effects]. Frequently, treatment effects were reported only narratively without providing absolute numbers, values, or frequencies.

**Conclusion:** Quality of materials differed, but none sufficiently fulfilled EBPI quality criteria. Unsatisfactory trial results concerning patient knowledge and patient involvement in decision-making may at least partially be explained by limitations of the provided materials. Future patient information should consider EBPI quality criteria.

## Introduction

Stroke is the leading cause of disability worldwide and affects quality of life (1). Despite increasing evidence for effective stroke prevention and treatment of stroke risk factors (2), stroke incidence and prevalence remain high (3). Efforts should focus on prevention and especially on secondary prevention, i.e., the prevention of further strokes in stroke patients.

Cardiovascular risk factors are a central target for stroke prevention approaches, but knowledge on risk factors in stroke patients is insufficient (4) and has not changed in recent years (5). Additionally, patients with increased stroke risk and stroke patients are often unaware of their risk status (5, 6). As a result, stroke patients may not engage in required preventive behaviors, e.g., blood pressure control, lifestyle modification, or medication intake for secondary prevention (7, 8).

Adherence to medical advice for secondary stroke prevention is poor, with one-quarter of stroke patients discontinuing prescribed medications 3 months after hospital discharge (9). A systematic review and meta-analysis of prospective epidemiological studies revealed that among cardiovascular diseases (CVD), including stroke, a substantial proportion of patients do not adhere adequately to cardiovascular medications. Approximately nine percent of all CVD events in Europe can be attributed to poor medication adherence (10).

Patients in different countries and settings claim multiple unmet educational needs concerning knowledge about the clinical aspects of stroke prevention and treatment (11). Overall stroke knowledge, as well as knowledge concerning secondary stroke prevention in patients with cerebrovascular diseases, is low to moderate (12).

Stroke patients want to be involved in treatment decisions and prefer an informed choice model of decision making (12). The use of decision aids may lead to informed choices and positively influence health behavior, by providing information on treatment options and supporting value clarification (13). Evidence-based patient information (EBPI) is an important part of decision aids. EBPI is based on systematic synthesis of the literature and communication of treatment effects using numerical data, presented in an understandable format (14). EBPI improves knowledge and increases satisfaction with the decision processes (14, 15). EBPI quality criteria defines how information should be presented (14) to allow for shared decision-making (15).

High-quality information constitutes the basis for shared decision-making, showing substantial potential to improve care (16–19). Consequently, increased patient involvement in decision-making may lead to behavioral changes, as we have shown in neurological patients with multiple sclerosis (20, 21).

In 2012, a Cochrane review analyzed the effectiveness of stroke patient information interventions (22). In total, 21 studies were included, covering a wide range of information materials (e.g., stickers, leaflets, booklets, videos, and audiotapes) from different sources (e.g., neurologists, pharmaceutical companies, and patient support groups). The review showed evidence that interventions addressing information provision can improve stroke knowledge. Actively involving patients may accelerate this effect (22).

Based on the Cochrane review, this study aims to analyze the quality of information materials for stroke patients tested in randomized-controlled trials (RCTs) by applying EBPI quality criteria (14).

## Materials and Methods

We updated the outdated search of the Cochrane review (22) that included RCTs, comparing information interventions with standard care, and used patient or carer knowledge as the primary outcome. We followed the search strategy of the Cochrane review and conducted searches in the following databases:

- Ovid MEDLINE and Epub Ahead of Print, In-Process & Other Non-Indexed Citations, Daily and Versions 1946 to July 2020
- Embase 1980 to July 2020
- CINAHL EBSCO from 1982 to July 2020
- PsycINFO 1806 to July 2020
- CENTRAL Issue 9 of 12, July 2020.

Titles and abstracts were reviewed by seven researchers and irrelevant studies were excluded. Full texts of the remaining studies were checked concerning the following inclusion criteria: randomized controlled trial, provision of stroke patient information for stroke patients, published in English, and knowledge assessed as the primary endpoint. Following the Cochrane review, we excluded trials in which information provision was only one component of a complex intervention. We initially aimed to focus on information materials addressing secondary stroke prevention only, which led to a small number of information materials. Therefore, we decided to evaluate all available materials addressing stroke patients. The software Rayyan (https://rayyan.qcri.org/) was used for study selection.

We added the results of our literature search to the studies included in the Cochrane review. We contacted authors of all studies, preferably via email, for a minimum of three times. If the first author was not available, senior authors or co-authors were approached. All authors were asked for information materials used and for consent to review these materials regarding EBPI criteria. Available information materials were analyzed using EBPI quality criteria (14) (for details see Table 1). We used a standardized data extraction sheet based on the item list (Table 1). Each quality item was rated as follows: 3 (yes/ complete/ good), 2 (in part/ incomplete/ satisfactory), or 1 (no/ unsatisfactory).

**Table 1:** Checklist of EBPI quality criteria (14).

	**Criteria**	**Short title**
1.	Is information given about the authors and their qualification?	Author
2.	Were patients, nurses, or relatives involved in preparation of the materials?	Co-worker
3.	When was the information material prepared?	Date
4.	Were conflicts of interest present and reported?	Conflicts of interest
5.	Is the financing of the information materials declared?	Financing
6.	Are the aims of the information materials given?	Aim
7.	Is the targeted group of the information materials specified?	Target population
8.	Are the information sources specified by the authors?	Sources
9.	Is information given about additional literature or links (internet)?	Links
10.	Are references and addresses or contacts (e.g., of a support group) named?	Contacts
11.	Is there a description of the disease evolution with and without treatment?	Natural course
12.	Do the authors name alternative treatments?	Alternative treatments
13.	If a treatment is mentioned, is information given about the effect of the treatment?	Treatment effect
14.	Is comprehensive information given about the benefit of the treatment or test?	Treatment benefit
15.	Is there comprehensive information given about the magnitude of the treatment effects?	Treatment effects, numbers
	1 = absolute risk reduction with reference parameter (1 of 100 Person)	1 = ARR
	2 = relative risk reduction, verbal frequencies (e.g., rare, often)	2 = RRR
	3 = no information on frequencies	3 = no
16.	Are side effects of the treatment described comprehensively and completely?	General side effects
17.	Is there a comprehensive statement of the frequencies of side effects or risk of harm?	Side effects, numbers
	1 = absolute risk increase with reference parameter (1 of 100 Person)	1 = ARR
	2 = relative risk increase, verbal frequencies (e.g., rare, often)	2 = RRR
	3 = no information on frequencies	3 = no
18.	Is information given about false-positive and false-negative results?	Diagnostic error
19.	Is the information meaningfully illustrated?	Graphical presentation
	1 = pictogram, pie chart, bar diagram, table, or figures	1 = complete
	2 = graphical presentation is incomplete/ insufficient legend	2 = incomplete
	3 = graphical presentations are missing	3 = missing
20.	Is the layout appropriate?	Comprehensive layout

*ARR, absolute risk reduction; RRR, relative risk reduction*.

## Results

**Figure 1:**
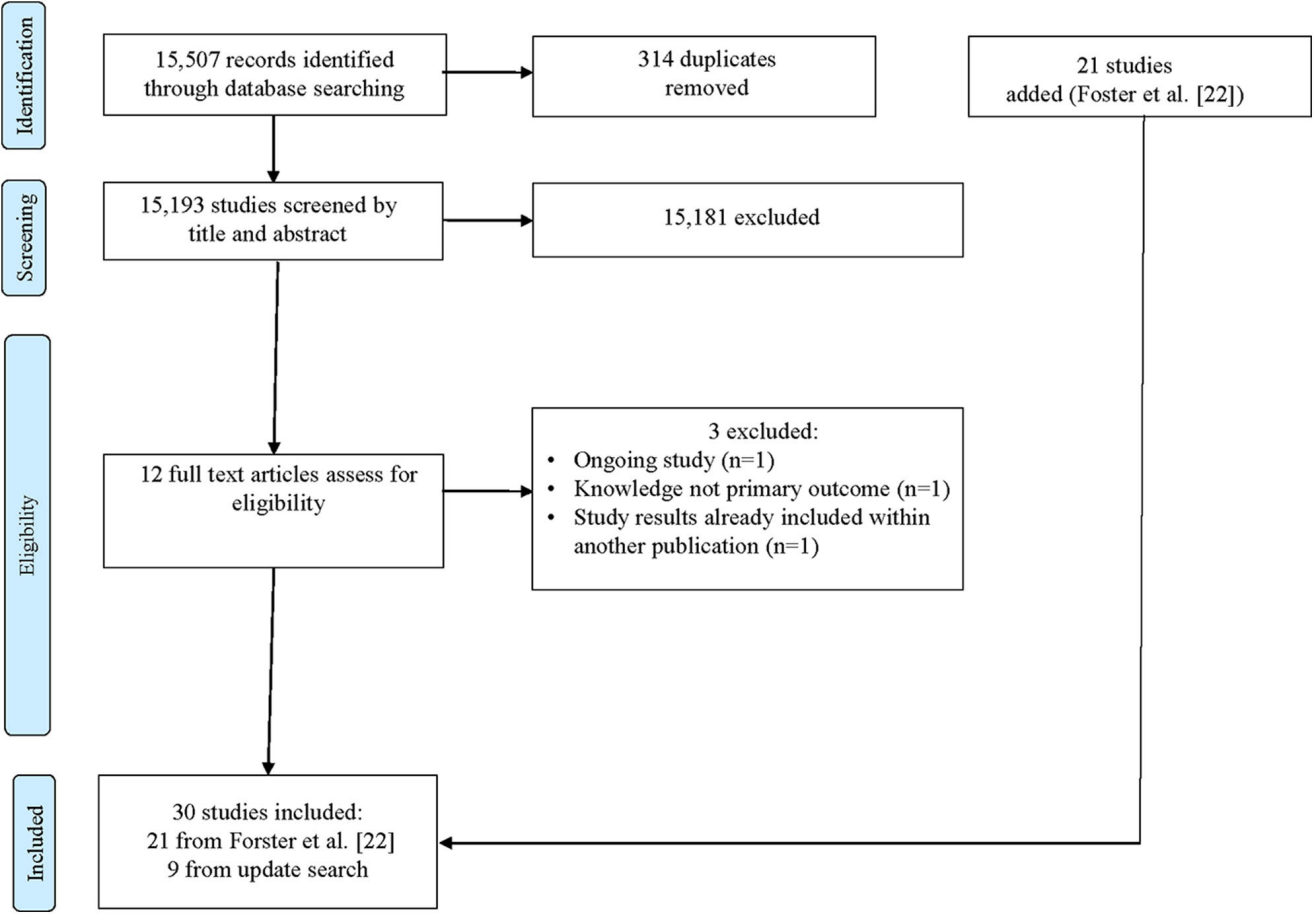
The flow diagram of the study selection following the PRISMA Guidelines (23). Identification shows the results of the updated systematic literature search following the Cochrane Review search strategy (22).

The search resulted in 15,193 citations without duplicates. 15,181 citations were excluded after title and abstract screening. In total, 12 full-text articles were assessed and nine studies were included (Figure 1). Those were added to the 21 studies already included in the Cochrane review. We contacted the authors of all 30 studies and received 16 replies. For 14 studies, authors could not be contacted (24–37). Of these, one study was excluded because materials were in Chinese (38). One study consisted of a multicomponent intervention, which we noticed after receiving the materials, and was therefore excluded (39). The remaining six authors were not able to send the materials, because they were not available anymore (40–45). Finally, eight (27%) studies provided materials that could be analyzed (Table 2).

**Table 2:** Assessment of information materials using criteria of evidence-based patient information (15).

**First Author**	**Draper (46)**	**Hoffmann (47)**	**Johnston (48)**	**Olaiya (49, 50)**	**Saal (51)**	**Sajatovic (52)**	**Smith (53)**	**Vormfelde (54)**
Year	2007	2007	2007	2016	2015	2017	2004	2014
Target population	Caregivers of aphasic patients	Stroke patients	Stroke patients	Stroke patients	Stroke patients	Stroke patients	Stroke patients	Patients with anticoagulation
*n*	31	133	203	142	265	38	170	319
Main topic	Effect of support, education and training on caregivers‘ burden and stress and communication	Effect of computer-generated tailored written information on satisfaction, knowledge, self-efficacy, depression	Effectiveness of a workbook-based intervention to enhance recovery, improve emotional outcomes	Effectiveness of a nurse-led intervention to improve management of risk factors	Effect of a stroke support service on physical function	Effect of a novel self-management intervention to reduce stroke risk	Effectiveness of an education program for patients and carers recovering from stroke	Effect of standardized patient education on knowledge and time within the target INR range
Material	Worksheets, overall 43 pages	Booklet, 34 chapters, 76 pages	Booklet, 14 chapters, 118 pages	17 leaflets, about 4 pages each	9 leaflets with different topics	Booklet, 39 pages	Ringfolder, 50 pages	Brochure, 16 pages
Intervention	Weekly groups or 4 weeks, each consisting of a 2-h caregiver intervention	Computer-generated tailored written information about stroke	Workbook-based intervention for 5 weeks after discharge	Individualized management program, involving nurse-led education, and management plan with medical specialist oversight	Outreach work, informational events, training sessions, online portal and written patient information	Self-management training, delivered in 1 individual and 4 group sessions	Specifically, designed stroke information manual, education meetings every 2 weeks	Video and information brochure

*INR, international ratio*.

### Characteristics of Included Studies (*n* = 8) (Table 2)

In the following, a short description of included studies is provided, sorted by the type of intervention.

#### Educational Programs

Draper et al. (46), Vormfelde et al. (54), Saal et al. (51), and Olaiya et al. (49) studied educational programs. Draper et al. (46) conducted an RCT with a wait-list design in Australia. Participants received a weekly educational program with four subsequent sessions, showing short-term effects on stress levels. Vormfelde et al. (54) performed a cluster RCT including a 1-h patient education session on oral anticoagulation with Phenprocoumon that included a video, a questionnaire, and an information brochure in Germany. The control group received the information brochure only. The intervention was feasible and improved knowledge. Saal et al. (51) conducted a stroke support service for post-stroke outpatients in Germany. The post-discharge stroke support service included outreach work (via telephone contact and home-visit), informational events, training sessions, an online portal, and written patient information. The control group received optimized standard care by written patient information (51). The service did not improve physical function, health-related quality of life, depression, somatization, or re-infarcts, but results suggest a lower overall risk of mortality in the intervention group (51). Olaiya et al. (49) included patients of the STAND FIRM RCT (55) evaluating the effectiveness of the nurse education component of the intervention in Australia. The intervention group received an individualized management program, comprising a chronic disease management plan and two home visits by nurses to provide tailored education for secondary prevention. The control group received the usual care. The study did not find any evidence for better knowledge of secondary prevention medications in the intervention group compared to controls.

#### Written Information Materials

Two studies applied written information materials (47, 53). Hoffmann et al. (47) compared a computer-generated tailored written education package in Australia to generic written information. Results showed improved satisfaction with the stroke information, but the program did not affect knowledge, self-efficacy, depression, or perceived health status. Smith et al. (53) conducted an RCT in England. Patients and caregivers in the intervention group received a specifically designed stroke information manual applied in bi-weekly meetings compared to the usual practice in the control group. Results showed no statistical difference in knowledge.

#### Self-Management Programs

Finally, Johnston et al. (48) and Sajatovic et al. (52) conducted self-management programs. Johnston et al. (48) performed an RCT in Scotland. Patients were allocated to a 5-week self-management intervention incorporating three information components and support and behavior change techniques after discharge or care as usual. The intervention group showed a better recovery compared to the control group. Sajatovic et al. (52) conducted a study assessing a self-management program to reduce secondary stroke risk in African-American men in the USA delivering a self-management training in one individual followed by group sessions over 3 months. Qualitative results suggested improved awareness of risk factors. For detailed data see Table 2.

### Quality of Information Materials (Table 3)

Quality ratings regarding EBPI quality criteria (14) revealed heterogeneous and often unsatisfactory results. Structural information about financing, authors, and co-workers were frequently met, while information about conflicts of interest and sources of information were often missing. In terms of content information, information about alternative treatments, treatment effects, and the natural course of the disease were frequently met. Information on treatment effects in (absolute) numbers was given only once, while reporting of side effects in numbers was not present in any information material (Table 3). Three materials were scored as moderate quality (reached at least 60% of the quality score, i.e., ≥36 of max. 60 points) (47, 53, 54). The other five materials were scored as low quality (<36 points).

**Table 3:** Quality rating based on established quality criteria for evidence-based patient information (18).

**Item**	**Short title**	**Draper (46)**	**Hoffmann (47)**	**Johnston (48)**	**Olaiya (49, 50)**	**Saal (51)**	**Sajatovic (52)**	**Smith (53)**	**Vormfelde (54)**	**Overall (range 8–24)**
1.	Author	3	2	2	1	1	2	2	3	16
2.	Co-worker	1	2	2	1	1	1	3	2	13
3.	Date	1	3	2	3	3	1	2	3	18
4.	Conflicts of interest	1	2	1	1	2	1	2	1	11
5.	Financing	1	2	1	2	3	1	3	1	14
6.	Aim	2	3	2	1	3	3	3	3	20
7.	Target population	1	1	3	2	3	3	2	3	18
8.	Sources	1	1	1	1	2	2	2	2	12
9.	Links	1	2	1	2	2	2	3	3	16
10.	Contacts	1	2	1	2	2	3	3	3	17
11.	Natural course	1	2	2	1	1	1	1	2	11
12.	Alternative treatments	1	2	2	2	1	2	1	2	13
13.	Treatment effect	1	2	1	2	1	2	1	3	13
14.	Treatment benefit	1	2	1	2	2	2	2	1	13
15.	Treatment effects, numbers	1	1	1	1	2	1	1	1	9
16.	General side effects	1	2	1	1	1	1	2	1	10
17.	Side effects, numbers	1	1	1	1	1	1	1	1	8
18.	Diagnostic error	–	–	–	–	–	–	–	–	0
19.	Graphical presentation	1	2	1	1	1	1	2	2	11
20.	Comprehensive layout	1	2	2	2	2	2	2	2	15
	Summary score (range 20–60)	20	36	28	29	34	32	38	39	

*3 = yes/complete/good*.

*2 = in part/incomplete/satisfactory*.

*1 = no/unsatisfactory*.

## Discussion

This literature review aimed to evaluate the quality of information materials for stroke patients. We evaluated RCTs from a Cochrane review (22) and updated the search. Although 30 studies were identified, information materials were only available for eight studies. All studies were RCTs addressing interventions aiming to inform stroke patients, but most studies had important methodological weaknesses. Analyses of the information materials revealed a profound lack of quality when applying EBPI quality criteria (15).

Results show important shortcomings in the investigated materials, especially regarding information on the natural course of the disease, which is essential to understand the possible benefit of a treatment (15) and crucial for informed decision-making, individual risk perception, preferences and values, physician expertise, and counseling (13).

Importantly, presentation of (absolute) numbers, values, or frequencies was mostly missing e.g., concerning treatment benefits or side effects. As both patients and physicians have difficulties in understanding risk data (56), providing only verbal descriptions does not meet state of the art risk communication (57).

The Cochrane review by Forster et al. (22) concluded that the best way to provide information to stroke patients is still unclear, as results show no consistent effect on knowledge and health behavior (22), although multiple unmet needs of patients regarding information provision for stroke have been reported (11). Considering the small number of available intervention materials and the often poor quality of the analyzed materials, it is not justified to conclude that patient information for stroke patients is not effective. We found the quality of information materials to be limited in all categories applied. Although aspects of reporting were at least met in some information materials, aspects referring to transparent reporting of results of treatment effects were presented inadequately throughout. Therefore, future interventions should use materials that adhere to the quality criteria of EPBI in order to be helpful for patients in making informed choices and being involved in shared decision-making. Specific focus should be placed on issues allowing patients to make informed decisions. Therefore, reporting of treatment effects using absolute differences instead of relative risks, reporting natural disease courses, and using graphical presentations adapted to the target group are needed to enhance informed decisions. Also, interventions should be described in detail, for example by using the TIDieR criteria (58), and materials should be made available. This conclusion is not new. In 1979, Ley et al. proposed that the content and form of patient information materials significantly impact on their effectiveness (59).

It has been argued that EBPI may disturb patients by communicating scientific uncertainties using absolute risks (19). However, most patients appreciate this transparent approach (19) and the use of absolute risks is generally recommended (60). We have repeatedly shown in neurological patients with multiple sclerosis that patients can understand and handle complex and even uncertain information (20, 61). Also, recent findings demonstrate that stroke patients, without cognitive impairment and/or aphasia, want to be involved in treatment decision-making and are able to understand basic statistical data without relevant differences to healthy controls (12).

The main strengths of our review are the use of established criteria for EBPI and the systematic search for RCTs on patient information interventions based on a Cochrane review. As a limitation, we were unable to evaluate most information materials, as there was no feedback from authors or materials were not available. This means that there is a risk of an inadvertent selection bias. Authors of studies who did not reply and those that could not provide materials may have not responded due to poor quality of the materials, leading to an overestimation of the quality of information materials. Another limitation is that the included information materials had different targets and were based on different concepts and sources of evidence. Some studies focused on basic stroke knowledge, while others aimed to support psychological aspects of rehabilitation after stroke.

In conclusion, we were able to evaluate eight information materials for stroke patients already tested in randomized controlled trials. Although some materials were rated considerably better than others, overall, materials did not meet the criteria of high-quality EBPI and therefore might not meet patients' needs as they fail to provide adequate information. Unsatisfactory trial results concerning patient knowledge and patient involvement in decision-making may at least partially be explained by the limitations of the provided materials. Future patient information materials should consider EBPI quality criteria.

## Data Availability Statement

All datasets generated for this study are included in the article/supplementary material.

## Author Contributions

AA made substantial contributions to conception and design, acquisition of data, analysis and interpretation of data, and drafting the article. AR made substantial contributions to conception and design, acquisition of data and analysis, and critically revised the article for important intellectual content. AS made substantial contributions to conception and analysis, interpretation of data, and also revised the article critically for important intellectual content. GT made substantial contributions to analysis, interpretation of data, and revised the article critically for important intellectual content. CH made substantial contributions to conception and design, analysis and interpretation of data and also participated in drafting the article and revising the article critically for important intellectual content. SK made substantial contributions to conception and design, acquisition of data, analysis and interpretation of data and participated in drafting the article and revising the article critically for important intellectual content. All authors contributed to the article and approved the submitted version.

## Conflict of Interest

AA received lecture fees from Bayer Vital. GT has received fees as a consultant or lecture fees from Acandis, Bayer Vital, Bristol-Myers Squibb/Pfizer, Boehringer Ingelheim, Daichii Sankyo, GlaxoSmithKline, and Stryker, and received a research grant from Bayer. The remaining authors declare that the research was conducted in the absence of any commercial or financial relationships that could be construed as a potential conflict of interest.
